# Sentinel Physicians for the Environment: A Chilean Perspective to Address Global Health and Climate Resilience

**DOI:** 10.3390/ijerph23030283

**Published:** 2026-02-25

**Authors:** Paolo Lauriola, Jaime Sepúlveda Cisternas, Lisa De Pasquale, Francesco Saverio Apruzzese, Xavier Maldonado, Olivia J. Brathwaite Dick, Yuri Carvajal

**Affiliations:** 1 European Public Health Alliance (EPHA), Rue de Trèves 49, 1040 Brussels, Belgium; 2 International Society of Doctors for the Environment (ISDE), 52100 Arezzo, Italy; lisadepa93@gmail.com (L.D.P.); francescosaverioapruzzese@gmail.com (F.S.A.); 3 Department of ISDE/FNOMCeO, Rete Italiana Medici Sentinella per l’Ambiente (RIMSA), 52100 Arezzo, Italy; 4 Department of Servicio de Salud Talcahuano, Talcahuano 4260000, Chile; jaime.sepulveda@hualqui.com; 5 Departamento de Medio Ambiente, Colegio Médico de Chile, Temuco 4780000, Chile; ycarvajal61@gmail.com; 6 Department of Biomedical, Metabolic and Neural Sciences, Faculty of Medicine, University of Modena and Reggio Emilia, Via Campi 287, 41125 Modena, Italy; 7 Facultad de Ciencias Médicas, Universidad Central del Ecuador, Quito 170521, Ecuador; fxmaldonado@uce.edu.ec; 8 Departamento de Enfermedades Transmisibles, Oficina de la Organización Panamericana de la Salud/Organización Mundial de la Salud, Santiago de Chile 7630412, Chile; brathwaiteo@paho.org

**Keywords:** sentinel surveillance, sentinel physicians for the environment, primary health care, climate change and health, One Health, Planetary Health, health equity, Latin America and Caribbean

## Abstract

**Highlights:**

**Public health relevance—How does this work relate to a public health issue?**

- Sentinel Physicians for the Environment (SPEs) provide an innovative and scalable approach for identifying and responding to environmental health threats—such as air pollution, heatwaves, wildfires, vector-borne diseases and antimicrobial resistance—directly within primary care, where most health needs are first detected worldwide.
- The model addresses a global gap in early detection of environmentally driven health risks by linking clinical observation with community-based monitoring across diverse health system settings.

**Public health significance—Why is this work of significance to public health?**

- Drawing on the emerging SPEs initiative in Chile and its relevance across Latin America, this work demonstrates that primary healthcare providers can act as frontline sentinels for climate-related and pollution-driven health impacts—an approach that is transferable to many other regions worldwide.
- The article advances a globally applicable framework for embedding environmental health capacity into Primary Health Care (PHC) systems, bridging the longstanding divide between environmental surveillance structures and everyday clinical practice.

**Public health implications—What are the key implications or messages for practitioners, policy makers and/or researchers?**

- Integrating SPEs into primary care can enhance preparedness, early warning and community resilience to environmental hazards in heterogeneous health systems across the world.
- The regional momentum generated around the 2025 Santiago Declaration offers a replicable model of governance and collaboration that countries outside Latin America may adopt to systematically incorporate environmental health into policy and practice.

**Abstract:**

Climate change and environmental degradation are intensifying health risks across Latin America, placing increasing pressure on primary health care (PHC) systems. Physicians working at community level are often the first to observe climate- and environment-related health effects, yet operational models that link clinical practice, environmental surveillance and community engagement remain insufficiently defined. This article adopts a policy-oriented narrative synthesis approach, drawing on peer-reviewed literature, policy documents, institutional records, memoranda of understanding, and outputs from professional seminars and stakeholder meetings conducted between 2024 and 2025 to develop an evaluable operational framework. Chile is examined as a case study, while the proposed framework is situated within a broader Latin American perspective. We conceptualise the model of Sentinel Physicians for the Environment (SPEs) as an operational framework embedded within PHC, structured around four core pillars: surveillance, prevention, communication and advocacy. The model clarifies how SPEs can contribute in practical terms to addressing major climate-related health threats, including heatwaves, air pollution, wildfires, vector-borne diseases, migration-related vulnerability, antimicrobial resistance and zoonotic risks. The Chilean experience illustrates feasible implementation pathways, distinguishing actions already undertaken, initiatives under development and proposed future steps. The SPE model offers a pragmatic and scalable approach to strengthening climate-resilient primary health care in Latin America. By leveraging existing PHC structures and community trust, SPEs can enhance early detection, risk communication and preparedness without requiring complex technologies or high financial investment, providing a transferable contribution to public health practice and policy, with clear implications for future evaluation.

## 1. Introduction

Climate change and environmental degradation represent unprecedented challenges for global public health. The health consequences range from the spread of vector-borne and zoonotic diseases to the exacerbation of respiratory and cardiovascular conditions, the amplification of mental health burdens, and the disruption of health systems and livelihoods [1]. Latin America, one of the most environmentally diverse and socially unequal regions of the world, is particularly vulnerable to the combined impacts of climate-driven extreme events, environmental pollution, and deep structural inequities. Recent analyses confirm that climate change profoundly affects Latin America and the Caribbean, not only through direct health impacts but also by driving migration and exacerbating social vulnerabilities [2]. These findings highlight the urgent need to link public health interventions with social protection and adaptation strategies.

Primary Health Care (PHC), as reaffirmed since the Alma-Ata Declaration, remains the cornerstone for addressing these evolving threats through proximity, continuity, and community engagement [3,4]. The distinction between General Practitioners (GPs) and Family Doctors (FDs) has been widely discussed. While GPs are generally defined as providers of non-specialist primary care at community level, FDs are rooted in family medicine, which conceptualizes the family rather than only the individual as the unit of care, reinforcing a holistic and relational approach to health. In this article, the term “General Practitioner” is used in a broad and generic sense for consistency and clarity. General Practitioners (GPs), traditionally focused on clinical care, are increasingly recognised as trusted actors who can bridge the gap between scientific evidence, community needs, and policy responses. Their professional ethos, rooted in ethics and social accountability, places them in a unique position to act not only as providers of care but also as “sentinels” who detect, interpret, and communicate environmental health threats.

As climate-related events, pollution clusters, and vector-borne diseases intensify across Latin America, PHC systems require new capacities to detect early warning signals and translate them into preventive and adaptive responses [5,6]. In order to address health risks amid the effects of global climate change, as suggested by OPS/OMS (2020), health systems will need to anticipate the existing and new challenges posed by climate change, prepare for them, and respond and recover quickly [7].

Within this context, the concept of Sentinel Physicians for the Environment (SPEs) has emerged as a practical and scalable approach to strengthen environmental health surveillance in primary care settings. SPEs are defined as family doctors, general practitioners, and other frontline clinicians trained to identify environment-related symptoms, interpret weak signals, detect clusters, and connect clinical observations with environmental exposures and community-level risks. Early experiences in Italy and other countries have demonstrated that SPE can support environmental monitoring, community education, rapid detection of environmental incidents, and collaboration with public health authorities [8,9,10]. Recent international analyses have reinforced the need to integrate environmental competencies into primary care to address climate-related health challenges, strengthen resilience, and bridge local observations with global health priorities [11,12,13].

Chile represents a particularly relevant setting for exploring and implementing the SPEs model. The country is simultaneously experiencing a high burden of environmental stressors—including air pollution, megadroughts, wildfires, mining-related exposures, and climate-sensitive infectious diseases—and possesses a strong tradition of community-oriented primary care through the *Modelo de Atención Integral de Salud Familiar y Comunitaria* (MAIS) [4,14,15,16], including the field of epidemiological surveillance. The Colegio Médico de Chile (COLMED) is the national medical association representing physicians in Chile. Beyond its role as a professional guild, it contributes to medical education, ethical regulation, and the defence of public health. COLMED has also become an institutional actor in health policy and international collaboration. Its Environment Department promotes physician engagement in environmental health, focusing on climate change, pollution, and ecosystem protection. In recent years, COLMED, local health services, and academic partners have initiated seminars, collaborative agreements, and pilot activities aimed at training SPE and integrating environmental health into clinical and community practice [17,18,19,20]. These steps highlight both the urgency of environmental health action and the opportunity to develop an operational model embedded in PHC.

This article aims to:

- Describe the emerging development of the Sentinel Physicians for the Environment model in Chile;
- Summarize the initiatives, collaborations, and early experiences generated through professional associations, health services, international cooperating agencies, and academic institutions;
- Contextualize the Chilean experience within international evidence on environmental health, PHC, and climate-resilience capacities;
- Discuss the potential for scaling SPE within Latin America as a contribution to strengthened environmental health preparedness, community resilience, and Planetary Health.

## 2. Chile’s Health System and the Role of Primary Care

Chile’s National Health System has long been recognised for its emphasis on equity and access, with a decisive role attributed to primary health care (PHC). Rooted in the principles of the Alma-Ata Declaration of 1978, which advocated for “Health for All” through community-based, preventive, and participatory approaches [3], Chile has gradually developed a system where family health centres (*Centros de Salud Familiar*) serve as the cornerstone of health service delivery. This structure has enabled health teams to establish close ties with local communities, fostering trust and allowing for early identification of both biomedical and social determinants of health [4].

During the 2000s, Chile adopted the MAIS, which placed families and communities at the centre of the health system. More recently, the country has strengthened this perspective through the *Estrategia de Cuidado Integral Centrado en las Personas* (ECICEP, 2020), a person-centred care strategy aimed at reconfiguring service delivery across the health network, especially primary health care, to better address multimorbidity and chronic conditions through a holistic, continuous and coordinated approach that incorporates the biological, psychological and social dimensions of individuals and their families [21]. This model prioritises preventive care, continuity, and intersectoral collaboration, explicitly recognising that health outcomes depend not only on clinical services but also on broader social and environmental conditions [4]. The MAIS framework provides a natural institutional entry point for addressing climate and environmental health challenges within PHC.

In practice, PHC centres across Chile have already been involved in environmental health monitoring and risk communication at the local level, including the surveillance of air pollution episodes in urban areas, provision of guidance during heatwaves, and preventive action against vector-borne diseases in peri-urban and rural zones. These experiences highlight the potential of physicians and healthcare teams to act as sentinels, connecting environmental signals with public health responses.

The relevance of this approach has been underscored by recent analyses from COLMED, which emphasise the need for a systematic assessment of environmental risks as part of health planning, with particular attention to climate-sensitive conditions such as respiratory, cardiovascular, and mental health conditions [22]. This diagnostic effort reflects a growing recognition that environmental health is not peripheral, but central to the sustainability and resilience of the Chilean health system.

For these reasons, Chile represents a fertile ground for the development of SPEs. By building upon existing PHC structures, leveraging the MAIS framework, and integrating environmental epidemiology into everyday medical practice, SPEs can reinforce both the preventive and community dimensions of the Chilean health system. Furthermore, their role as trusted professionals embedded within local communities positions them to strengthen resilience against climate change impacts and to act as intermediaries between affected communities, policymakers, and scientific networks.

**Figure 1:**
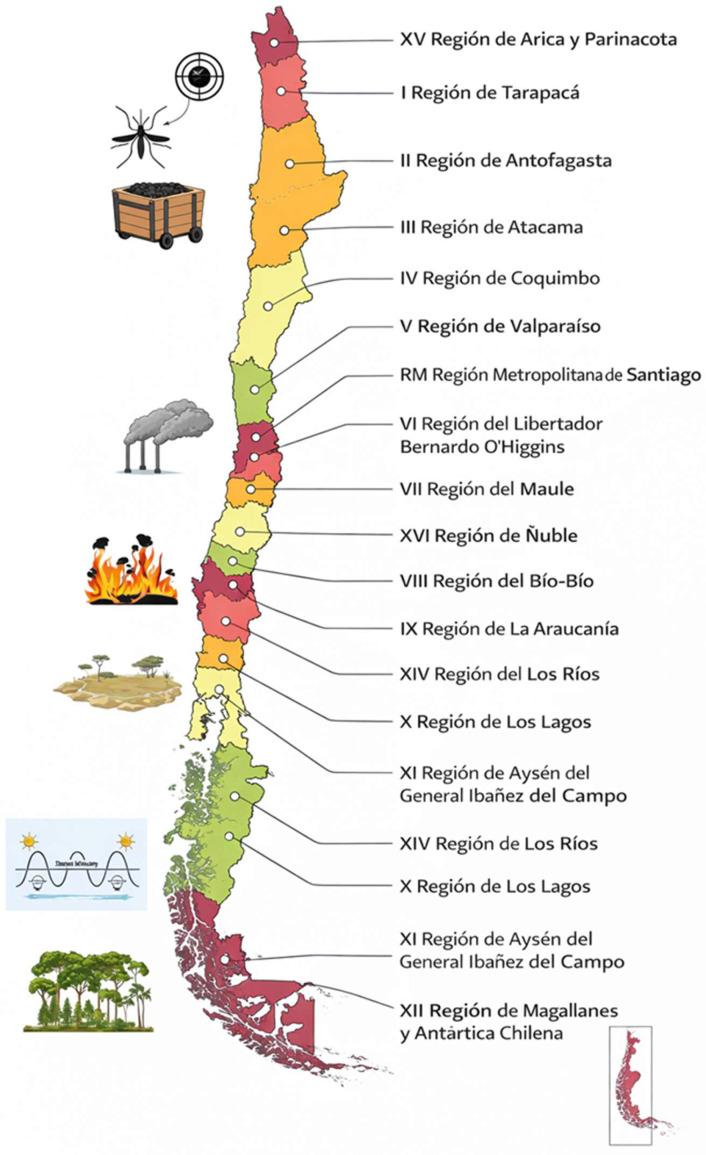
Environmental-health risks in Chile. The figure presents a schematic overview of Chile’s administrative regions, with illustrative colours and icons highlighting major environmental and climate-related health challenges discussed in the text. The map shows the 16 administrative regions of Chile, from the northernmost Región de Arica y Parinacota to the southernmost Región de Magallanes y de la Antártica Chilena. The country spans approximately 4300 km from north to south, and the schematic map has an approximate scale of 1:16,000,000. The figure is intended for illustrative purposes only. Readers requiring greater detail, the original image can be easily accessed via the provided link. Non-English labels indicate the official Spanish names of Chile’s administrative regions. Source: Wikimedia Commons (https://es.wikipedia.org/wiki/Archivo:Mapa-chile.svg; Accessed on 23 September 2025); modified.

Environmental health risks in Chile can be identified both at the macro-geographical scale and at the local level. At the national level, risks are unevenly distributed: vector-borne diseases in the north, air pollution in Santiago, drought and wildfires in the central-southern regions, and climate variability in the south. In addition, specific local risks, such as those linked to mining industries, industrial pollution, and climate-related hazards, represent critical challenges for population health. Air pollution remains one of the most critical public health challenges in Chile, particularly in Santiago. A bibliometric study confirmed the consolidation and growth of scientific evidence in this area [14]. These overlapping exposures highlight potential areas of action for SPEs. Evidence from case studies, such as the impacts of mining tailings in Chañaral [23] and the increased respiratory diseases among children living near mining areas [16], further underline the importance of integrating local industrial and climatic exposures into health strategies (Figure 1).

## 3. International and Latin American Reference Experiences

The idea of SPEs builds upon diverse international and regional experiences that have progressively integrated health, environment, and community action [8]. In Latin America, where structural inequities and environmental vulnerabilities converge, such models have proven particularly relevant.

One of the most emblematic recent initiatives has been the Healthy Brazil Programme (*Programa Brasil Saudável*), launched in 2024 as an intersectoral strategy uniting 14 ministries. The programme explicitly incorporates environmental and social determinants into public health strategies, aiming to eliminate 11 socially determined diseases and 5 vertically transmitted infections by 2030. By aligning health with sectors such as housing, education, and environment, Healthy Brazil demonstrates how health systems can simultaneously address neglected diseases, pollution, and climate-related risks, while reinforcing equity, social participation, and community resilience [24]. This initiative builds on the broader tradition of the Brazilian Unified Health System (SUS), which has long prioritised universality, equity, and social participation as guiding principles of public health policy [25].

At the urban level, air pollution control in megacities such as Rio de Janeiro and São Paulo has provided valuable lessons for environmental epidemiology and health policy. Long-term studies have documented the health burden of particulate matter (PM) and other pollutants, showing significant associations with hospitalisations for respiratory conditions and related economic costs, thereby informing both local interventions and national legislation [26]. These experiences illustrate the potential role of SPEs in systematically collecting, interpreting, and communicating environmental health data from the frontline of communities.

### Implications for Chile

The Brazilian experience offers strategic lessons for the ongoing development of Sentinel Physicians for the Environment (SPEs) in Chile. First, its strong intersectoral governance framework, linking primary care, environmental surveillance, social protection and community participation, demonstrates that environmental health functions can be institutionalised within PHC without creating parallel systems. Second, Healthy Brazil shows how structured integration between ministries, municipalities and community actors enables early warning, environmental risk monitoring and communication with vulnerable population groups, similarly to the collaborative architecture envisaged for SPEs in Chile.

For Chile, these experiences illustrate both feasibility and sustainability: environmental anamnesis, participatory risk mapping and local surveillance can be incorporated into existing MAIS structures if supported by multidisciplinary teams, municipal environmental departments and strong community participation. Finally, the Brazilian case shows that primary care can be an operational entry point for climate and environmental resilience, providing a realistic benchmark for future evaluation, curricular planning and governance pathways in Chile.

Another critical body of evidence comes from community-based participatory research (CBPR), which has gained prominence in Latin America as a tool to empower communities, reduce inequities, and integrate local knowledge with scientific inquiry. By engaging community members directly in the definition of priorities, design of interventions, and interpretation of results, CBPR has proven effective in addressing environmental health challenges ranging from pesticide exposure to urban sanitation [27]. GPs could play a crucial role as facilitators of participatory processes that link epidemiological surveillance with community empowerment.

Regional institutions have stressed the need for coordinated approaches, as shown by Pan American Health Organization’s (PAHO) recent guidelines on tungiasis [28], a neglected tropical disease shaped by environmental and climatic factors [29,30]. This highlights the urgency of integrating such determinants into health strategies, where GPs in community-centred frameworks can act as both care providers and agents of environmental monitoring and policy change. In 2020, PAHO published “Climate Change for Health Professionals: A Pocket Book”, to increase knowledge on the subject and the capacity of health systems to anticipate, prevent, and prepare in order to consistently provide high-quality health services in a world with a rapidly changing climate [7].

## 4. Climate Change, Environment, and Emerging Health Risks in Latin America

Latin America is experiencing the compounded effects of climate change, deforestation, urbanisation, and persistent social inequities. These dynamics interact to generate a spectrum of health risks that are increasingly evident in epidemiological trends and public health practice.

### 4.1. Climate Change and Systemic Vulnerabilities

Climate change is projected to amplify health inequities across the region, particularly affecting children, youth, and socially disadvantaged groups. As noted by ECLAC and UNICEF, the disproportionately low allocation of climate finance to children reflects a structural weakness in addressing health equity, leaving a highly vulnerable population group insufficiently protected against climate-related risks [6].

Ethical dimensions are increasingly discussed, with physicians identifying climate change as both a moral and professional challenge. As already mentioned, a national survey of US-based physicians confirmed a growing awareness of inequitable impacts and a willingness to engage in sustainable practices [5]. This resonates in Latin America, where professional organisations are beginning to frame climate action as part of medical responsibility.

### 4.2. Wildfires, Deforestation, and Respiratory Health

Extreme wildfires have become one of the most visible consequences of climate change in South America. The 2017 “megafire” in Chile destroyed more than 570,000 ha, caused 11 deaths, and resulted in damages exceeding USD 350 million [31]. More recently, during February 2023, drought and persistent heatwaves fuelled wildfires that burned over 420,000 ha in central Chile [15]. In early February 2024, another catastrophic fire event caused 131 deaths and destroyed more than 14,000 homes in the Valparaíso region, particularly in Viña del Mar and Quilpué, affecting over 21,000 people [32,33]. Within the framework of the “Wildfire Recovery Plan in Valparaíso for Mental Health and Psychosocial Support (SMAPS)” implemented by PAHO, in collaboration with the Ministry of Health and with the financial support of the Government of Canada, a series of social dialogues were developed with first-response health personnel and members of organized civil society in 2025. Communities reported widespread mental-health impacts, while available psychological care was insufficient to meet demand [34]. These recurrent events illustrate how climate-driven extreme fires increasingly interact with social vulnerabilities. A recent global analysis projected that climate-driven wildfires could cause over one million premature deaths annually by the end of the century, reshaping global health inequalities [35]. Preparedness and resilient health systems are therefore essential, given both the direct health effects (injuries, burns, respiratory conditions) and the indirect ones (continuity of care).

Deforestation across the Amazon basin and the Andean foothills has exacerbated wildfire risks, reduced water security, and increased exposure to smoke and particulate matter. In addition to acute events, long-term exposure to air pollution remains a major driver of premature mortality in the region [36].

### 4.3. Migration and Social Instability

Climate-induced migration represents another emerging challenge. Prolonged droughts, floods, and hurricanes are driving displacement across Central America, the Caribbean, and the Andean region. Migrants often face precarious living conditions, disruption of health services, and heightened exposure to infectious diseases. The Lancet Regional Health-Americas has emphasised that climate-driven migration interacts with pre-existing inequalities and weakens health protection systems [2]. In Chile, border health services received the massive influx of migrant groups, who arrived with various communicable and chronic diseases, malnutrition, exposed to extreme cold or heat, and above all, with major mental health challenges. These dynamics directly affect the social fabric and require adaptive responses from health systems.

### 4.4. Infectious Diseases, Vectors, and One Health Risks

Vector-borne diseases illustrate the direct biological links between climate change and health in Latin America. Dengue has reached unprecedented levels, with more than 12.6 million cases and 7700 deaths reported in 2024 across the region, particularly in Argentina, Brazil, and Colombia [37]. Chikungunya and Zika continue to pose threats in tropical and subtropical areas, with periodic outbreaks affecting both urban and peri-urban populations [38].

Moreover, climate variability affects the ecology of vectors such as *Aedes aegypti*, facilitating the expansion of arboviruses into new areas, including temperate regions [39]. Beyond arboviruses, zoonotic spillovers and antimicrobial resistance (AMR) linked to parasitic diseases are increasingly recognized as pressing One Health challenges, with evidence of resistant pathogens in South American wildlife and companion animals [40,41], underscoring the need for integrated surveillance and control strategies.

### 4.5. Operational Role of Sentinel Physicians for the Environment in Major Hazard Scenarios

To clarify the practical relevance of SPEs, each of the major climate- and environment-related threats described above corresponds to specific, recurring operational functions within the SPE model. During heatwaves and acute air-pollution episodes, SPEs can detect early clinical signals (e.g., heat-related symptoms, respiratory exacerbations), provide tailored counselling to vulnerable individuals, and facilitate timely communication with public health and environmental authorities. In contexts of water stress or contamination, SPEs may recognise clusters of gastrointestinal or dermatological symptoms and activate municipal services for hazard mitigation. Regarding migration, they contribute to identifying emerging vulnerabilities and environmental risks disproportionately affecting displaced populations. For vector-borne diseases, SPEs can detect unusual patterns of febrile illness and collaborate with PHC teams and entomological surveillance units. In relation to antimicrobial resistance, SPEs support antibiotic stewardship, monitor recurrent infections and inappropriate prescribing patterns, and promote rational antimicrobial use at the community level. Under conditions suggestive of zoonotic spillover, SPEs may identify atypical clinical presentations, refine differential diagnoses, and coordinate with veterinary and public health agencies.

Beyond surveillance, SPEs exert a direct influence on individual clinical trajectories by promoting preventive, climate-sensitive behavioural changes, such as adopting energy-efficient practices, shifting towards plant-forward diets, engaging in active or low-emission mobility, reducing waste, and choosing safer housing locations in hazard-prone areas. These actions strengthen awareness, adaptive capacity and self-protection within households. During emergencies, SPEs also support preparedness and risk communication, assist patients in navigating emergency services, and help minimise health impacts—consistent with evidence highlighting the value of interdisciplinary training for disaster and emergency response.

#### 4.5.1. Heatwaves

SPEs integrate basic environmental anamnesis and vulnerability assessment into routine consultations (age, isolation, chronic illness, housing insulation, access to cooling) and communicate risk signals to municipal health authorities to support targeted alerting for frail elders and chronically ill individuals. Expected benefits may include earlier outreach and improved continuity of care during peak events. Notably, Chilean academic and public health discourse has increasingly emphasised the importance of continuity of care and community-based primary health care as key adaptive strategies to extreme heat events, recognising that models grounded in long-term patient–community relationships are better suited to identify vulnerability, support behavioural adaptation, and reduce heat-related health impacts [42]; within this framework, the Sentinel Physicians for the Environment (SPE) approach has been explicitly discussed as a potentially valuable tool for climate-change adaptation in the health sector.

**Proposed illustrative indicators for future pilot evaluation may include:**

1. The proportion of high-risk patients (e.g., older adults, people with chronic conditions, socially isolated individuals) receiving documented heat-vulnerability screening during officially declared meteorological heat alerts;
2. The mean time elapsed between the identification of clusters of heat-related symptoms in primary care consultations and notification to municipal or regional health authorities.

#### 4.5.2. Wildfire Smoke

SPEs support clinical triage of respiratory complaints, advise on exposure minimisation (ventilation, filtration, protective behaviours), and identify vulnerable households. Observational data are shared with municipal epidemiology units to enable rapid community messaging and preparedness.

**Proposed illustrative indicators for future pilot evaluation may include:**

1. The number of respiratory exacerbation cases in which wildfire smoke exposure is explicitly documented in the clinical record;
2. The timeliness of community risk communication following SPE reporting of clustered respiratory symptoms during wildfire smoke episodes.

#### 4.5.3. Urban Air Pollution

SPEs incorporate preventive counselling into chronic disease management (COPD, asthma, cardiovascular risk), detect clusters of acute exacerbations, and transfer contextual observations through primary-care reporting systems to municipal environmental authorities.

**Proposed illustrative indicators for future pilot evaluation may include:**

1. The proportion of patients with chronic respiratory or cardiovascular conditions receiving documented air-pollution–related preventive counselling during high-pollution episodes;
2. The number of clinically identified symptom clusters communicated from primary care to municipal environmental or public-health authorities.

#### 4.5.4. Dengue and Vector-Borne Expansion

SPEs contribute to early identification of atypical febrile cases in previously low-risk areas, facilitate participatory risk mapping, and support micro-environmental surveillance (water accumulation, waste, mobility). These signals strengthen early warning and community-level action.

**Proposed illustrative indicators for future pilot evaluation may include:**

1. The time interval between first presentation of atypical febrile cases in primary care and notification to epidemiological surveillance services;
2. The number of community or household-level risk-mapping actions initiated following SPE signal detection in previously low-risk areas.

#### 4.5.5. Migration-Related Vulnerability

SPEs document environmental determinants affecting mobile or displaced families (heat exposure, housing, water quality, occupational risk), support referral continuity, and inform municipal health and social-protection services.

**Proposed illustrative indicators for future pilot evaluation may include:**

1. The proportion of migrant or displaced patients with documented assessment of environmental and housing-related risk factors during primary care consultations;
2. The number of referrals or alerts transmitted from primary care to municipal health and social-protection services in response to identified environmental vulnerabilities.

#### 4.5.6. Antimicrobial Resistance and Zoonotic Spillover

SPEs record environmental exposure patterns (waste management, animal proximity, community mobility) and contextualise febrile or gastrointestinal complaints, supporting dialogue with veterinary and environmental agencies.

**Proposed illustrative indicators for future pilot evaluation may include:**

1. The frequency of documented environmental or animal-exposure histories in patients presenting with recurrent infections or atypical clinical patterns;
2. The number of clinical–environmental observations shared with veterinary, environmental, or public-health agencies to support One Health surveillance and dialogue.

These mechanisms are developmental and do not represent formal evaluation outputs. The information flow descriptions should be interpreted as illustrative operational pathways, while the indicators listed above are proposed examples for future pilot monitoring and evaluation rather than already-measured performance metrics.

## 5. The Rationale for Sentinel Physicians for the Environment (SPEs)

As outlined in the Introduction, this section provides a more systematic rationale for considering GPs as trusted professionals embedded within their communities.

The evidence presented above illustrates how climate change and environmental degradation in Latin America translate into multiple, interconnected health risks. These include the direct consequences of extreme weather events and wildfires, the indirect impacts of migration and social disruption, and the biological amplification of infectious diseases. Addressing such complex challenges requires health professionals who can operate at the interface between clinical practice, public health, and environmental surveillance.

GPs hold a unique social position as trusted professionals embedded within communities. Their day-to-day contact with patients allows them to detect early signals of changing epidemiological patterns, such as unusual clusters of respiratory disease during pollution episodes, heat-related illnesses during heatwaves, or vector-borne infections in previously unaffected areas. If systematically supported and connected to public health institutions, such observations can serve as an invaluable early warning system. This sentinel role is particularly relevant in Latin America, where health inequities and environmental vulnerabilities converge.

The rationale for SPEs builds on foundational dimensions. First, environmental epidemiology in clinical practice, enabling physicians to contextualise health problems within environmental exposures. Second, the promotion of community engagement and participatory methods, drawing from the traditions of community-based participatory research (CBPR), which have proven effective in addressing pesticide drift, air pollution, and other environmental health challenges across the region [27]. Third, the strengthening of intersectoral collaboration, positioning physicians as intermediaries between scientific institutions, policymakers, and local populations.

By combining these dimensions, SPEs can contribute to:

**Surveillance and prevention**, through systematic monitoring of climate- and pollution-related health effects. *Example from Chile*: In municipal primary care centres, early SPE pilot experiences have incorporated basic environmental anamnesis and clinical–environmental triage into routine consultations, enabling the recognition of heat-related symptoms, respiratory exacerbations during wildfire smoke episodes, and gastrointestinal clusters potentially linked to water contamination. These observations are recorded through standard primary-care documentation systems and can be used to inform municipal epidemiology units. This approach has been piloted in the Biobío Region as part of the 2024–2025 “*Protocolo de vigilancia epidemiológica de efectos en salud por eventos de calor extremo*”, coordinated by the Regional Ministerial Secretariats (SEREMI de Salud), where sentinel health establishments systematically monitor heat-related clinical presentations during declared meteorological alerts, linking primary-care observations with regional epidemiological assessment [43].

**Community empowerment**, by sharing knowledge and mobilising local action to mitigate risks. *Example from Chile*: Participatory risk-mapping exercises in family health centres have allowed SPEs and community partners to identify micro-environments of concern (urban heat pockets, poorly ventilated housing, proximity to industrial emissions, waste accumulation). These activities support dialogue with vulnerable households and municipal services, and have strengthened preparedness for heatwaves and wildfire smoke.

**Policy translation** by transforming clinical and community observations into evidence that informs health and environmental governance. *Example from Chile:* through emerging SPE coordination mechanisms with municipal environmental-governance platforms, primary-care teams have begun to contribute clinical–environmental insights to local preparedness plans for extreme heat, respiratory peaks, and wildfire smoke. This collaboration supports more integrated risk communication and targeted outreach to frail patient groups.

These three foundational dimensions enable the SPEs approach conceptually. The effectiveness of SPEs also depends on the quality of the networks in which they are embedded, requiring fluid communication, participatory decision-making, and continuous monitoring of performance.

### 5.1. Conceptual Architecture of Sentinel Physicians for the Environment

**Figure 2:**
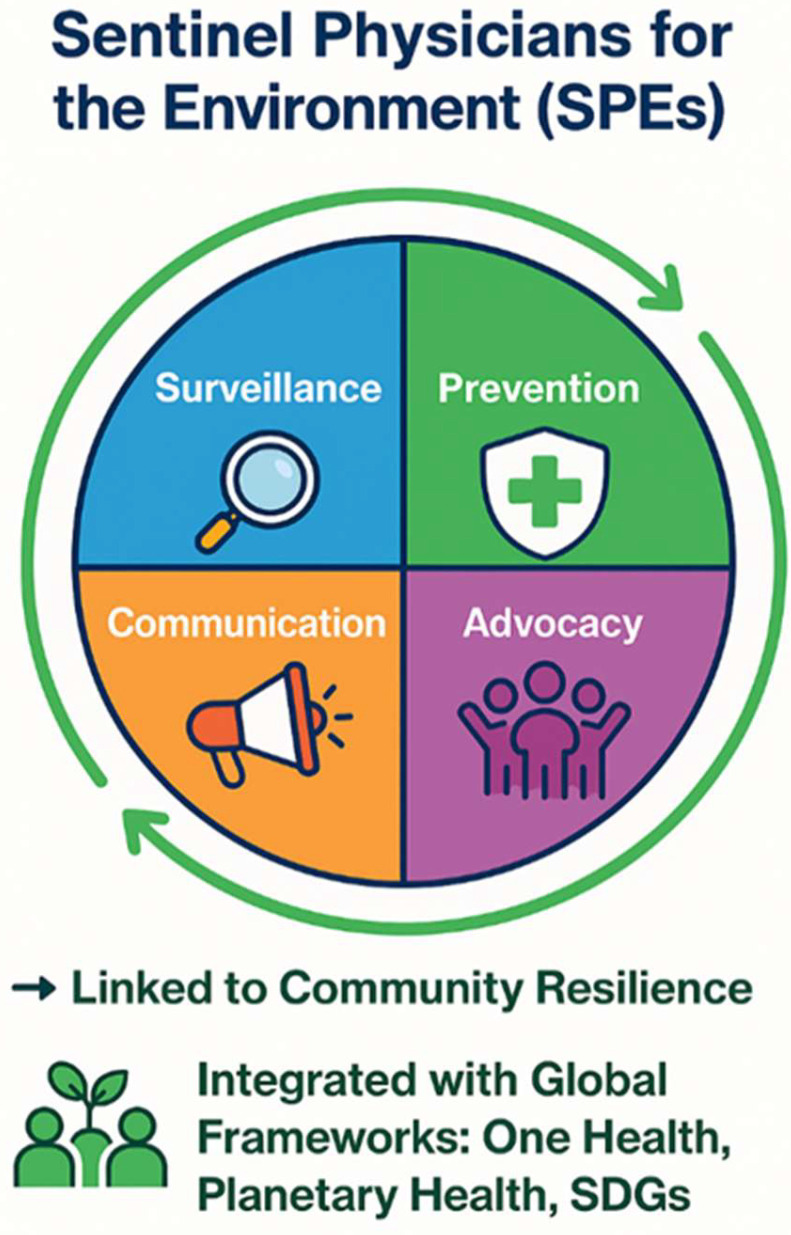
Conceptual model of Sentinel Physicians for the Environment (SPEs). Diagram of the proposed conceptual model for SPEs, based on four core functions: surveillance, prevention, communication, and advocacy. These functions are interconnected with community resilience and aligned with global frameworks such as One Health, Planetary Health, and the Sustainable Development Goals (SDGs). The model illustrates how primary care can act as a bridge between community-level observations and national and international health strategies. Arrows represent dynamic and bidirectional interactions among the components of the model.

Building on these dimensions, the SPEs model is operationalised through four core functions: surveillance, prevention, communication, and advocacy into a single framework, adding value to global strategies such as One Health, Planetary Health, and the Sustainable Development Goals (Figure 2). The model illustrates how primary care can act as a bridge between community-level observations and national and international health strategies. The 4 pillars of the model are outlined below.

#### 5.1.1. Surveillance

The function of Surveillance positions SPEs as crucial nodes for environmental epidemiology, integrating clinical observation with environmental tracking.

The Chilean initiative, aligning with previous experiences like the Italian Network of Sentinel Physicians for the Environment (RIMSA), shows how general practitioners and paediatricians can be integrated into environmental surveillance.

The clinical observations provided by SPEs can thus be directly integrated with environmental surveillance data. For these reasons, the sentinel physician network enables public health departments to conduct epidemiological environmental surveillance.

#### 5.1.2. Prevention

Prevention focuses on proactive measures to mitigate environment related health risks, often through intersectoral and community-based approaches.

Prevention is carried out on two levels: by individual SPE, who can implement preventive measures for the patients they examine, and at a higher level by the sentinel physician network. The network enables the public health department to obtain up-to-date data on which to base preventive campaigns.

#### 5.1.3. Advocacy

The Advocacy function transforms evidence gathered by physicians into policy influence through the SPEs network, bridging local action with global climate concerns.

This function aligns with the ethical responsibilities of physicians in responding to climate-related health challenges.

SPEs are also engaged in advocacy by promoting awareness and preventive behaviours among their patients.

#### 5.1.4. Communication

Effective Communication is essential for governance within the SPE network, ensuring that insights from the frontline reach decision-makers and the public.

Effective communication ensures that both the public and decision-makers receive clear, timely, and actionable information to support informed choices and coordinated responses.

The SPEs model must go hand in hand with proper education of physicians.

Table 1 provides a systematic, pillar-by-pillar operationalisation of the Sentinel Physicians for the Environment (SPE) framework. For each of the four core pillars—surveillance, prevention, communication, and advocacy—the table explicitly distinguishes key operational constraints and resource requirements, reporting and information flow pathways, and proposed illustrative indicators for future pilot evaluation. The table illustrates how planning and reflection tools such as Strengths, Weaknesses, Opportunities and Threats (SWOT) analysis, prioritisation criteria, and the “5 Ws and How” approach can be applied in a differentiated manner across the four SPE pillars, rather than transversally, in order to support operational clarity and evaluability [44,45].

As shown in Table 1, for the surveillance pillar, the framework highlights the need for sufficient institutional capacity within primary care settings, including the availability of structured documentation tools and coordination mechanisms with municipal and regional epidemiological services. The reporting pathway is explicitly articulated, tracing the flow of clinical observations from the consultation room to municipal health offices and onward to regional surveillance units. Illustrative indicators focus on timeliness and signal generation, such as the interval between cluster identification and notification and the number of environment-related reports originating from primary care.

The prevention pillar is characterised by operational constraints related to competing clinical priorities, limited consultation time, and the availability of local preventive services. Its reporting pathway centres on the clinical encounter, where preventive counselling and risk communication may lead to referral to health, social, or environmental services. Proposed indicators therefore focus on coverage and documentation, including the proportion of high-risk individuals receiving preventive counselling and the systematic recording of environmental risk factors in primary care records.

For the communication pillar, key constraints include the risk of misinformation, the need for coordinated institutional messaging, and linguistic or cultural barriers within communities. Reporting and information flow are conceptualised as bidirectional processes linking SPE signal detection with municipal communication units and public information channels. Evaluation considerations emphasise the timeliness and reach of risk communication, reflected in indicators such as the number of community advisories issued and the speed of communication during environmental or climate-related events.

The advocacy pillar operates within a distinct set of constraints, including political sensitivity, institutional mandates, and the need to protect individual clinicians from personal exposure. Reporting pathways therefore rely on the aggregation of SPE observations through professional associations and public health institutions, enabling structured policy dialogue at municipal or regional levels. Illustrative indicators focus on policy uptake and institutional influence, such as the inclusion of SPE-generated evidence in planning documents or the frequency of policy-relevant reports informed by sentinel observations.

Planning and reflection tools (e.g., SWOT analysis, prioritisation criteria, the Eisenhower matrix, and the ‘5 Ws and How’ approach) are proposed as supportive instruments to be applied within each pillar, complementing the operational and evaluative structure presented in Table 1.

Overall, this pillar-specific articulation strengthens the operational credibility and evaluability of the SPE framework by demonstrating how distinct functions can be realistically implemented, monitored, and refined within primary health care systems. In this sense, the SPE model provides a pragmatic translation of Planetary Health principles into medical and public health practice, explicitly linking human health, environmental sustainability, and social justice.

In conclusion this rationale resonates with the broader Planetary Health perspective, which emphasises the inseparability of human health, ecosystems, and social justice [1]. In this sense, SPEs embody a practical translation of Planetary Health principles into medical and public health practice.

## 6. Methodological Approach and Sources of Evidence

This paper adopts a narrative synthesis and policy analysis approach, rather than a systematic review. This is intentionally not a systematic review, but a qualitative synthesis of policy documents, scientific literature, and practical experiences generated through collaboration between public health actors in Latin America. Its objective is to conceptualize a Sentinel Physicians for the Environment (SPE) framework, drawing from existing public health structures, primary-care governance, and environmental-health vulnerabilities in the Latin American region.

### 6.1. Data Sources and Identification Strategy

The Analysis Relied on Three Main Families of Sources

**Scientific literature** retrieved through searches in PubMed, Scopus, and SciELO between 2015 and 2025, using combinations of the following keywords: *environmental surveillance*, *primary health care*, *sentinel networks*, *climate change and health*, *community health workers*, *participatory epidemiology*, and *Latin America*. Sources were included if they provided insights into environmental health practice, sentinel-based approaches, or the role of primary care in environmental exposure assessment. Documents in English, Spanish, Portuguese, and Italian were analysed to ensure regional representation.

**Internal documentation and experiential outputs** generated through ongoing collaboration between COLMED, ISDE Italy, PAHO, WHO-HQ and local governments. These included seminar agendas, minutes, presentations, memoranda of understanding (MoUs), internal reports, community field notes, and pilot program descriptions. These internal documents were reviewed using a qualitative-thematic strategy, identifying recurring operational elements relevant to SPE implementation, such as community mapping, participatory risk identification, and clinical–environmental triaging.

**Participatory evidence and experiential knowledge** from multidisciplinary seminars and workshops carried out in Chile, Ecuador and Brazil between 2023 and 2025. These events involved physicians, community health workers, municipal authorities, NGOs, family-health educators, and environmental specialists, and sought to assess the feasibility and cultural appropriateness of an SPE approach in real primary-care settings.

### 6.2. Analytic Process

Thematic Synthesis was Performed Across all Documents to Identify

- Environmental-health functions already embedded in primary care;
- Gaps in surveillance, early warning and communication;
- Opportunities for SPE deployment at different levels (clinic, municipality, region).

On this basis, the analysis informed the development of a Sentinel Physicians for the Environment (SPE) framework, grounded in existing public health structures, primary-care governance, and environmental-health vulnerabilities in the Latin American region. This article does not present original epidemiological surveillance data. Instead, it develops a conceptual and operational model based on a qualitative synthesis of scientific literature, institutional documentation (seminar minutes, MoUs, municipal resolutions), and experiential knowledge generated through ongoing collaborations in the Chilean and Latin-American public health context. Accordingly, its purpose is analytical and developmental rather than evaluative, and all proposed mechanisms should be interpreted as operational pathways rather than measured outcomes.

### 6.3. Limitations

This work is primarily conceptual and does not report empirical health outcome data or quantitative exposure measurements. Its purpose is to develop an operational framework based on existing evidence, field experiences, and governance practices. While the results are grounded in real institutional experiences from COLMED and its partners, they should not be interpreted as programme evaluation outputs. Future studies should collect empirical indicators to evaluate SPEs performance in terms of early warning, clinical risk triage, cross-sector coordination, and communication with vulnerable groups.

It proposes an operational and organisational framework for SPEs, based on a narrative synthesis of scientific literature, institutional documents, policy materials, and documented professional experiences. The aim is to support conceptual clarity, implementation pathways and future evaluation, rather than to report measured outcomes. This methodological positioning is intended to ensure transparency and to align the scope of the article with its policy-oriented and practice-informed objectives.

## 7. Implementation Pathways for Chile and Latin America

The development of SPEs in Chile is evolving through institutional engagement, multisectoral dialogue, and capacity building. To ensure conceptual clarity, we distinguish three levels of maturation of the model: implemented actions, initiatives currently under development, and aspirational proposals designed to support regional-scale adoption.

### 7.1. What Has Already Been Implemented

Activities in this category are already initiated and institutionally anchored and have been carried out through established collaboration between COLMED, municipal health directorates, local universities, international agencies, and community organisations.

Implemented actions include:

The delivery of **environmental-health seminars** for family physicians and primary-care teams in multiple regions, addressing clinical–environmental risk assessment, participatory mapping, heat-related illness management, wildfire exposure, industrial emissions, and community resilience.

In 2024, COLMED organised a cycle of environmental-health seminars for family physicians and primary-care teams, addressing climate-induced migration, community vulnerability, industrial pollution, wildfire exposure, and sustainability practices in the health sector (COLMED, *Seminarios sobre salud, medio ambiente y cambio climático*, Santiago 2024) [46]. These events were delivered with participation from municipal authorities, academic institutions and civil society organisations, reinforcing interdisciplinary dialogue and strengthening institutional awareness of environmental determinants of health.

The establishment of **formal collaboration agreements (MoUs)** between COLMED and professional, municipal and academic partners to advance environmental-health surveillance and community preparedness.

Between 2024 and 2025, COLMED signed Memoranda of Understanding with ISDE and EPHA, formalising collaboration on environmental-health surveillance, training, community preparedness and scientific advocacy [17,18]. These MoUs provide institutional anchoring and technical support for the future development of SPEs in Chile and ensure alignment with international environmental-health frameworks

The involvement of **municipal authorities** and civil society organisations to integrate environmental risk detection into community health boards, family-health centres and participatory planning platforms.

Since 2020, COLMED’s public health journal *Cuadernos Médico Sociales* has dedicated two permanent sections to environmental and Planetary Health—*Sobrevivencia de Chile* and *Cuadernos Botánico Sociales*. These sections promote scholarly reflection, interdisciplinary dialogue and coordination between health professionals, academic partners and civil society, reinforcing the institutional culture required to sustain SPE development and community engagement.

PAHO, through its Virtual Campus of Public Health, had offered self-paced online courses for health professionals, including the “Basic Course in Environmental Epidemiology” and the “Health Impact Assessment for Air Pollution with AirQ+” course, among others. With the support of COLMED, a course will be created to provide convenient and timely online access to the pocketbook “Climate Change for Health Professionals”.

Since 2025, collaboration between COLMED and primary care has been operationally strengthened through the integration of the national Association of Primary Care Physicians into COLMED’s Department of Environment. This organisational step has enabled joint training pathways, shared seminar design, and more systematic involvement of primary care teams in environmental-health capacity building and SPE pilot activities.

#### Public Health Relevance

These implemented actions have strengthened the **situational awareness** of primary-care structures, improved the recognition of environmental determinants by clinicians, and introduced concrete mechanisms for **community dialogue**, especially in wildfire-prone or highly polluted urban areas.

### 7.2. Initiatives in Active Development

This category refers to organisational steps that have been formally initiated, with stakeholder engagement, curricular planning, or pilot-scale implementation underway.

**Figure 3:**
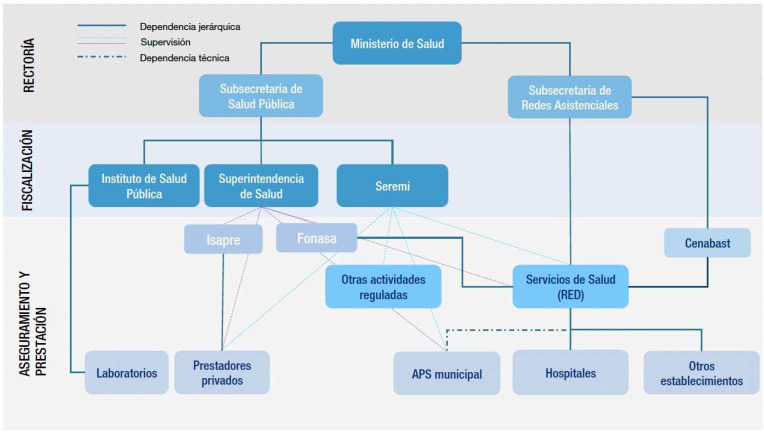
Organization and functioning of the Chilean National Health System [47]. The Ministry of Health (MINSAL) provides overall stewardship through two Undersecretariats, the Public Health and Healthcare Networks and the Regional Ministerial Secretariats (SEREMI). Twenty-nine territorial Health Services manage hospitals and municipal primary health care (PHC) centres. Dependent agencies include the Institute of Public Health (ISP), the National Health Fund (FONASA), the Central Supply Centre (CENABAST), and the Superintendence of Health. The private sector is represented by ISAPREs (private insurers) and accredited providers. Solid arrows indicate routine hierarchical relationships, dashed arrows indicate supervisory or regulatory functions, and dotted arrows indicate technical support or coordination pathways.

Figure 3 illustrates the conceptual positioning of SPEs within existing primary-care structures, municipal environmental governance and community health platforms in Chile. It visually clarifies how clinical teams, community actors and municipal authorities interact in operational terms, supporting early detection, participatory risk mapping and targeted communication for vulnerable groups.

Figure 3 also illustrates the functioning of the system: solid lines indicate hierarchical dependence, dashed lines supervisory functions, and dotted lines technical guidance or coordination. Currently under development:

- The creation of structured SPE training curricula for family physicians, nurses, environmental-health workers and community teams, developed with technical input from COLMED, ISDE-Italy, WHO-HQ and PAHO.
- The formalisation of SPE working groups, including local primary-care centres, municipal epidemiology teams, environmental departments and academic partners, to coordinate field implementation and evidence gathering.
- The integration of SPE activities into routine clinical documentation systems (electronic patient registers, risk dashboards, community indicators), enabling early detection of clusters, recurrent exposure, or vulnerable households.
- The strengthening of municipal environmental-governance platforms, enabling primary care to contribute to local risk mapping, preparedness for heatwaves or wildfire smoke, and targeted alerting for vulnerable patient groups.
- On 26 August 2025, the *Servicio de Salud* Talcahuano, together with municipal authorities, academic partners and community organisations, organised a seminar dedicated to climate resilience, environmental determinants of health and migrant vulnerability [19]. The seminar facilitated interdisciplinary dialogue, strengthened stakeholder awareness and supported the early formation of institutional networks relevant for future SPE planning (Servicio de Salud Talcahuano, Jornada sobre cambio climático, migración y salud, 2025).

At this stage, these activities remain at the level of curricular planning, institutional design and early coordination. They do not constitute operational implementation or evaluable surveillance outputs. Their purpose is developmental and exploratory, forming a structured pathway for future testing and evaluation.

### 7.3. Aspirational/Proposed Actions

These actions are not yet implemented and should not be interpreted as established practice or evaluated programmes.

Future proposed actions include:

- The establishment of a National School of Sentinel Physicians for the Environment, coordinated by COLMED and national universities, to consolidate training, accreditation and multidisciplinary research.
- The creation of sentinel community health centres, where SPEs collaborate systematically with schools, fire departments, urban-development authorities, emergency platforms and environmental labs.
- The regionalisation of the SPE approach in Latin America, supported by PAHO and WHO-HQ, promoting cross-country learning, shared indicators, comparative risk analysis, and coordinated capacity-building.
- The consolidation of a Latin-American governance platform promoting early warning, environmental-health knowledge transfer, community resilience, and transboundary policy coordination.

### 7.4. Regional Declaration and Institutionalisation

A major step forward in this direction was the approval of the ***Santiago Declaration on Air Quality and Public Health in Latin America***, formally adopted on 28 November 2025 [48]. The declaration represents a regional commitment to advance environmental health, community resilience, and cross-sector governance through the active involvement of primary health care and sentinel providers.

During the Ibero-Latin American Conference on Air Quality and Public Health (Santiago, 30–31 October 2025) [49], multiple stakeholders highlighted the strategic value of involving primary health care and sentinel clinicians in environmental surveillance, early warning and community resilience. These contributions informed the drafting of the Santiago Declaration, which explicitly recognises sentinel functions within primary care as an operational component of environmental-health governance in Latin America. The Declaration therefore provides political endorsement for the progressive institutionalisation of SPEs, strengthening regional legitimacy for pilot implementation, capacity building and cross-country learning

The Declaration was developed through regional collaboration involving practitioners, municipalities, academic partners and public-health organisations from Chile, Ecuador, Brazil and other LAC countries, with the technical support of WHO-HQ and PAHO.

The process involved about 100 participants, both in-person and remotely, and led to the creation of a multidisciplinary governance group dedicated to monitoring implementation, promoting SPE capacity-building, and facilitating country-to-country knowledge transfer.

The Santiago Declaration will be publicly disseminated through the websites of COLMED, PAHO, ISDE Italy and EPHA, and serves as a regional roadmap to advance sentinel-based environmental surveillance within primary care.

This document does not claim that these aspirational actions are already implemented or evaluated; rather, they represent a structured and collective proposal grounded in experiences already observed in Chile and in LAC.

### 7.5. Education and Capacity Building for Sentinel Physicians for the Environment in Chile and Latin America

Building on these initiatives, the potential establishment of a National School of Sentinel Physicians for the Environment is being explored as a developmental pathway rather than an implemented action.

The School would institutionalise training in environmental epidemiology, climate resilience, and community engagement, creating a permanent platform for capacity building. Conceived as a structural follow-up to the MoUs with ISDE and EPHA, it could also serve as a reference model for other Latin American countries.

#### 7.5.1. Foundational Principles

Education and training are central to consolidating and expanding the SPE model. Structured programs in environmental, planetary, and community health for medical and public health professionals have been widely recognised as essential [11]. International experiences show that when family physicians are directly engaged, Planetary Health principles can be effectively integrated into pre- and post-graduate education, clinical governance, and public health decision-making [12].

As highlighted in the Latin American context, community-centred training programmes in environmental and climate-sensitive care have demonstrated that family physicians can effectively bridge clinical practice, environmental surveillance, and social vulnerability analysis. This perspective aligns with the call for integrating environmental content into medical curricula advanced by Mantilla et al. in their regional capacity-building initiative on climate and health within the Andean countries [50].

Structured programmes in environmental and Planetary Health for medical professionals have been widely recognised as essential for preparedness and resilience. International experiences show that when family physicians are directly involved in educational innovation, Planetary Health principles can be integrated into undergraduate medical teaching, postgraduate training, and continuing education. A concrete illustration is provided by the recent Chilean academic contribution exploring the relevance of Planetary Health in medical training in the Anthropocene [20]. This approach underscores the importance of climate awareness, environmental determinants of health, and interdisciplinary collaboration in medical education.

#### 7.5.2. Educational Rationale

The SPE educational proposal seeks to consolidate a professional profile capable of:

- Integrating environmental anamnesis into routine consultations;
- Recognising clusters of environmentally linked morbidity;
- Supporting intersectoral preparedness and community engagement;
- Translating clinical observations into actionable early warning and risk-communication processes.

These functions are particularly relevant in Latin America, where climate change, environmental degradation, migration, and health inequities intersect, often affecting vulnerable populations. The recent regional declaration on air quality and health highlights the centrality of primary care in environmental vigilance and adaptation strategies. As emphasised by Franco et al. in their Brazilian experience with community-based environmental health research, participatory methods, interdisciplinary learning, and locally anchored educational activities are effective mechanisms for strengthening resilience and collective awareness [51].

#### 7.5.3. Structured Curriculum and Competency Targets

The proposed SPE curriculum includes:

- Environmental epidemiology and clinical–environmental risk assessment;
- Planetary Health concepts and links with public and individual health;
- Preparedness and risk-communication competencies for heatwaves, wildfire smoke, vector-borne disease expansion, and environmental emergencies;
- Participatory tools such as community mapping, household vulnerability assessment, and micro-environmental surveillance;
- Intersectoral governance and collaboration with municipal, environmental, and veterinary institutions;
- Ethical responsibilities of physicians in climate-related and environmental-health decision making.

Educational delivery may be organised across undergraduate programmes, postgraduate family-medicine pathways, and continuing professional development. The proposal also emphasises sustained mentoring and field-based learning through collaboration with municipal services and primary care governance platforms. International collaboration is a strategic asset: PAHO, WHO-HQ, ISDE Italy, and regional universities can contribute scientific expertise, accreditation frameworks, and harmonised evaluation criteria.

#### 7.5.4. Institutional Development and Operationalisation

Advancing SPE capacity-building will also require structured collaboration among health professionals, the Ministry of Health, whose *Estrategia de Cuidado Integral Centrado en las Personas* (ECICEP) promotes a comprehensive, person-centred and community-oriented approach, and the Association of Faculties of Medicine (ASOFAMECH), responsible for the training of medical and allied health professions. In addition, a coordinated platform involving the national network of Schools of Public Health, COLMED, and other professional associations could further harmonise the four operational pillars of the SPE framework and promote standardised training, accreditation pathways and interdisciplinary governance.

A School of Sentinel Physicians for the Environment is proposed as a national academic-professional platform. It would develop curricula, certify competencies, coordinate interdisciplinary teaching teams, and maintain close collaboration with municipal epidemiology, environmental surveillance, and community-resilience programmes. Its institutional partners may include COLMED, public universities, local health authorities, and regional governance actors. Monitoring indicators may include:

- The number of trained professionals;
- Adoption of environmental anamnesis in primary care;
- Local environmental risk-mapping activities;
- Integration of sentinel observations into municipal surveillance systems;
- Strengthening of community preparedness and continuity of care during emergencies.

Such an initiative builds upon the tradition of family and community medicine in Latin America, where interculturality, participatory decision-making, and territorial health perspectives are deeply rooted. In Ecuador, the MAIS model already requires environmental and social determinants to be integrated into situational health analysis, participatory community mapping, and interdisciplinary planning. The experience shows that incorporating environmental components into primary care curricula is both feasible and aligned with existing governance approaches. These insights mirror the principles developed by the Bolivian *Salud Familiar Comunitaria Intercultura* (SAFCI) model and the Peruvian “*Modelo de Cuidado Integral por Curso de Vida*”, where community participation, environmental determinants, and local resilience are systematically embraced.

#### 7.5.5. Regional Vision

The consolidation of SPEs education across Latin America is not an isolated initiative but a regional proposal grounded in shared environmental vulnerabilities and common public-health challenges. A regional working group, supported by PAHO, WHO-HQ, and civil society partners, can harmonise competency frameworks, define cross-country indicators, promote mutual learning, and facilitate policy translation. This approach encourages local adaptation while promoting regional coherence and scientific comparability.

## 8. Discussion and Policy Implications in Chile

### 8.1. Comparison with Other Sentinel Systems

The use of sentinel physicians to detect early signals of population-level health threats has a long and well-established history. One of the earliest modern examples was the Epidemic Observation Unit established in Birmingham in the mid-1950s, where general practitioners systematically reported unusual clinical events to identify emerging epidemics in the community [52].

Among infectious diseases, influenza surveillance represents the most consolidated and globally structured sentinel system. For several decades, general practitioners and family doctors have reported influenza-like illness (ILI) on a weekly basis, providing early warning of seasonal epidemics and supporting preparedness decisions. At the global level, the World Health Organization’s Global Influenza Surveillance and Response System (GISRS) coordinates data from National Influenza Centres and reference laboratories, integrating clinical and virological information for risk assessment and vaccine strain selection [53].

At the regional and national level, the European Centre for Disease Prevention and Control (ECDC) compiles information from national sentinel networks [54], while in the United States the CDC’s ILINet collects reports from outpatient practices to monitor influenza activity [55].

The public health relevance of sentinel approaches to influenza has been emphasised in international reflections on how carefully placed sentinel sites can enhance global early warning capacity [56].

In the Asia–Pacific region, initiatives such as the Alumni for Global Surveillance network (AGSNet) have contributed to the early detection of avian influenza and other emerging infections [57], whereas in Australia the Australian Sentinel Practices Research Network (ASPREN) [58] has provided more than three decades of ILI surveillance, often acting as an anticipatory indicator for trends observed later in the Northern Hemisphere [59].

Zoonotic and vector-borne diseases offer additional, highly relevant examples of sentinel networks. Systems monitoring West Nile virus, dengue, chikungunya and other arboviral infections typically combine clinical, entomological and environmental data, illustrating the importance of multi-source information, geographical specificity and structured communication between clinicians, laboratories and public health authorities [37,38,60].

In Latin America, Brazil has implemented multisectoral approaches in which primary healthcare units, local laboratories and environmental surveillance services collaborate to monitor dengue, chikungunya, tungiasis and other climate-sensitive or neglected conditions, showing that PHC-embedded sentinel systems can function effectively in complex socio-environmental settings [24,30].

Recent scoping reviews of sentinel physician networks indicate that most documented experiences concern infectious diseases—particularly influenza and zoonoses—while initiatives explicitly involving physicians as sentinels for environmental health remain comparatively limited. Nevertheless, a series of documented cases shows that frontline clinicians often recognise environmentally driven health problems—such as pesticide-related events, industrial emissions, persistent organic pollutants and other pollution-related hazards—before they are formally detected by institutional systems.

Within this broader tradition, Sentinel Physicians for the Environment (SPEs) can be seen as both a continuation and an extension of sentinel practice. Building on prior frameworks for involving family doctors and paediatricians in environmental health surveillance, SPEs are conceived as primary-care clinicians trained to detect environmentally driven clinical signals, identify clusters potentially related to exposure, and collaborate systematically with public health and environmental agencies [8,19].

Unlike classical sentinel systems, which mainly target infectious pathogens, SPEs networks explicitly address pollution-related, climate-related and multi-source environmental hazards. This positions SPEs as a complementary innovation that preserves the strengths of traditional sentinel approaches proximity, early detection capability and structured information flow, while expanding their scope to contemporary environmental determinants of health.

### 8.2. Integration of SPEs Within Latin American PHC Models

Beyond Chile, several Latin American PHC models already incorporate structural features that align naturally with SPE functions. In Ecuador, the national MAIS requires environmental exposure assessment during clinical anamnesis and mandates community situational analyses using participatory tools such as “talking maps” and community actor mapping [61]. These activities offer clear operational entry points for SPEs to detect environmental hazards, strengthen environmental health literacy, and coordinate local responses.

Bolivia’s SAFCI model (*Salud Familiar Comunitaria Intercultural*) strongly integrates environmental and intercultural dimensions within PHC, providing another coherent framework for SPEs implementation [62].

Similarly, Peru’s Comprehensive Health Care Model (MCI) includes environmental and social determinants of health across the life course [63], and Colombia has embedded environmental health as a core dimension within its national PHC-oriented framework [64].

These experiences suggest that integrating SPEs within the Chilean MAIS, and within analogous PHC models in the region, is not an additional burden but a natural expansion of existing responsibilities. In many countries, PHC facilities already enter routine clinical and community data into national health information systems, allowing SPE observations to support public health planning, interinstitutional coordination, and early warning. This perspective also supports the potential development of broader Sentinel Environmental Health Teams, reflecting the multi-professional nature of PHC in the region.

### 8.3. SPEs Conceptual Model in Chile

Building on Chilean and international experiences, a conceptual model for SPEs can be outlined to illustrate how physicians bridge local realities with global health frameworks (Figure 2).

The Chilean experience with SPEs, promoted by COLMED through seminars and institutional partnerships, illustrates how primary care can address the health impacts of climate change and environmental degradation. This initiative must be understood in the broader context of global and regional efforts to integrate Planetary Health principles into health systems.

Benton L. et al. (2025) [1] argue for the necessity of adopting a Planetary Health perspective in guidance for complex interventions on climate and health, highlighting the value of cross-disciplinary and community-based approaches. Similarly, Hantel. et al. (2025) [3] emphasise the ethical responsibilities of physicians in responding to climate-related health challenges.

Latin America provides a particularly relevant setting for this discussion. Batista, C. et al. (2024) [2] show how climate change and migration are deeply intertwined in the region, producing new vulnerabilities that challenge health systems. UNICEF and ECLAC (2025) warn that up to 17.9 million children in Latin America and the Caribbean could be pushed into poverty by 2030 if mitigation and adaptation policies are not accelerated, underscoring the urgency of integrating child-sensitive climate and health policies [6].

At the same time, Brazil has pioneered innovative models for integrating public health, community participation, and environmental protection. The *Healthy Brazil* initiative highlights how intersectoral approaches can strengthen resilience [24]. Participatory research experiences further demonstrate that community engagement and health system responsiveness can be mutually reinforcing [54]. These lessons are directly relevant to the Chilean context, where SPEs could combine clinical practice with community empowerment.

Vector-borne diseases and zoonoses represent another urgent dimension. Recent dengue epidemics illustrate how climate variability, urbanization, and social vulnerability combine to amplify health risks across the region [65]. Arbovirus dynamics are directly linked to climate variability and land use. Broader structural risks have also been highlighted in global analyses of zoonotic threats [66,67]— A recent study in the Brazilian Amazon confirmed that extreme weather and ENSO events are major drivers of malaria transmission, with highly heterogeneous impacts across states and ecological contexts, underscoring the need for fine-scale, climate-informed early warning and control strategies [68]. Experiences such as these reinforce the potential role of SPEs in Chile, where clinical observation could be integrated with environmental surveillance to strengthen early warning capacity.

The region is also heavily affected by climate-related disasters. In Chile, successive megafires (2017 and 2023–2024), compounded by a prolonged “megadrought”, devastated ecosystems, reduced water availability, and aggravated social and health vulnerabilities [31,33,69]. These compound events underscore the importance of preparing local health systems for resilience, where SPEs can serve as connectors between community-level observations and institutional responses.

The Chilean initiative is not isolated, but instead builds upon international collaborations with ISDE and EPHA, aligning with previous experiences in Italy. The Italian Network of Sentinel Physicians for the Environment (RIMSA) showed how general practitioners and paediatricians can be integrated into environmental surveillance [8]. More recently, a participatory study in Verona documented pesticide contamination in residential areas near vineyards and demonstrated how GPs can collaborate with citizens to produce environmental evidence [70]. Indoor air pollution has also emerged as a crucial issue, as shown in a consensus by Italian experts [71].

The COVID-19 pandemic confirmed the relevance of primary care in responding to global environmental threats. Lessons from Italy demonstrated that primary and community healthcare are essential in building resilient systems [9]. The experience of Italian GPs during the pandemic further illustrated the intersection between environment and health [10]. The need for integrating GPs into surveillance frameworks has been reinforced by Lauriola P. et al. (2024), who argued that GPs play a crucial role in comprehensive health surveillance [72].

Finally, the role of GPs in bridging global climate concerns with local action has been described extensively. Taken together, these experiences from Chile, Italy, and Latin America suggest that SPEs can represent a replicable model for strengthening environmental epidemiology and public health resilience. By combining clinical observation with community engagement and institutional collaboration, SPEs can act as a bridge between local realities and global health priorities. This approach not only reinforces the ethical role of physicians but also provides a practical pathway for implementing Planetary Health principles at the frontlines of care.

From a policy perspective, SPEs embody a replicable model for strengthening adaptive capacity within health systems. They integrate surveillance, prevention, communication, and advocacy into a single framework, adding value to global strategies such as One Health, Planetary Health, and the Sustainable Development Goals. Their policy relevance extends to climate finance, ensuring resources reach frontline services; to equity, by targeting vulnerable groups; and to intersectoral action, by fostering collaboration among health, environmental, and social policies. Nevertheless, challenges remain in terms of resources, political will, and training infrastructure. At the same time, opportunities are emerging to scale up the Chilean experience across Latin America, supported by regional cooperation, COLMED’s leadership, and the recent “Santiago Declaration on Air Pollution in Latin America”.

The Chilean experience suggests that Sentinel Physicians for the Environment (SPEs) can represent a replicable model not only for health surveillance but also for training and advocacy. By integrating these three dimensions, surveillance, education, and policy implications, SPEs can reinforce resilience at the intersection of health systems, communities, and territories.

At the policy level, SPEs align with global frameworks such as One Health, Planetary Health, and the Sustainable Development Goals. Their contribution could be particularly relevant for advancing intersectoral climate policies, ensuring equitable access to climate finance, and promoting resilience-oriented healthcare systems in Latin America

In response to recent academic critiques, it is important to situate the SPE model within an explicit and reflexive discussion of the operational limits and opportunities of One Health and Planetary Health frameworks.

While the concepts of One Health and Planetary Health have gained significant traction in public health discourse, an expanding body of academic literature has highlighted both their conceptual value and the challenges associated with their operationalisation. Several authors note that, if applied in an abstract or overly technocratic manner, these frameworks risk oversimplifying the complexity of environment–health interactions and insufficiently addressing social and economic determinants of health, particularly in low-resource settings and contexts marked by structural inequities [73,74].

At the same time, critical analyses do not reject the relevance of One Health; rather, they underscore the need for context-sensitive and health-system-embedded implementation strategies. In low- and middle-income countries (LMICs), barriers such as fragmented governance, limited intersectoral coordination, and resource constraints have been identified as major obstacles to the effective implementation of One Health strategies [75]. However, emerging evidence suggests that these limitations can be partially addressed when One Health principles are operationalised through Primary Health Care (PHC)–centred approaches. Recent work has shown that PHC offers a pragmatic entry point for One Health implementation, particularly when family physicians and community-based health teams are engaged as integrative actors at the interface of human, animal, and environmental health. By embedding One Health functions within routine clinical practice, community engagement, and local surveillance, PHC-based models can facilitate early detection, risk communication, and intersectoral collaboration without requiring highly specialised infrastructure [76]. In this sense, PHC-oriented approaches help translate One Health from a normative framework into an operational strategy that is more feasible and sustainable in LMIC contexts.

Within this critical yet constructive perspective, the Sentinel Physicians for the Environment (SPE) model can be interpreted as a PHC-grounded operationalisation of both One Health and Planetary Health principles, explicitly designed to address the practical challenges identified in the literature while maintaining a strong focus on social vulnerability, community participation, and local governance [77,78,79].

### 8.4. Implementation Challenges and Sustainability Considerations

#### 8.4.1. Workload, Time Constraints, and Burnout

The implementation of Sentinel Physicians for the Environment (SPEs) within primary health care inevitably raises concerns regarding workload, time constraints, and the risk of professional burnout among already overstretched primary care teams. In many Latin American settings, general practitioners and family physicians operate under conditions of high patient volumes, limited consultation time, and administrative burden. For this reason, the SPEs model is not conceived as an additional task layer, but rather as an integrative approach that embeds environmental awareness and sentinel functions within existing clinical workflows. Environmental anamnesis, early signal recognition, and contextual documentation are framed as extensions of routine clinical reasoning, aligned with continuity of care and community-oriented practice, rather than as parallel reporting obligations.

#### 8.4.2. Legal Authority, Governance, and Accountability

A second critical challenge concerns governance arrangements, legal authority, and accountability for physician reporting of environmental health observations. In most countries, including Chile, primary care physicians do not operate under a specific legal mandate to report environmental hazards in the same way as notifiable infectious diseases. The SPEs model therefore relies on structured collaboration with municipal health authorities, epidemiological surveillance units, and environmental agencies, using existing reporting channels wherever possible. Clear institutional agreements and protocols are required to clarify roles, responsibilities, and data stewardship, ensuring that clinical observations contribute to public health decision-making without exposing individual clinicians to legal uncertainty or inappropriate liability.

#### 8.4.3. Political and Economic Resistance, and Independence

Environmental health surveillance and early warning may generate political and economic sensitivities, particularly when signals point to pollution sources, industrial activities, or land-use practices with commercial relevance. Potential resistance from affected economic actors represents a recognised barrier to the effective translation of environmental health evidence into policy action. Within this context, maintaining scientific independence, transparency, and institutional legitimacy is essential. The SPEs framework emphasises collective reporting through professional and public-health institutions rather than individual advocacy, thereby reducing personal exposure for clinicians and strengthening the credibility of environmental health signals through aggregation, peer validation, and alignment with public-sector governance structures.

#### 8.4.4. Data Infrastructure and Information Systems

The transformation of clinical observations into actionable environmental health intelligence depends on the availability and interoperability of data infrastructure. Many primary care information systems are not currently designed to capture environmental exposure data in a structured manner, nor to facilitate real-time linkage with environmental monitoring or municipal surveillance platforms. The SPEs model therefore acknowledges existing limitations in digital infrastructure and emphasises incremental integration, using adapted clinical records, simple coding practices, and local dashboards where feasible. Investment in interoperable information systems remains a prerequisite for scaling SPEs activities beyond pilot settings and for supporting systematic monitoring and evaluation.

#### 8.4.5. Funding, Stewardship, and Long-Term Sustainability

Long-term sustainability represents another major challenge. While early SPEs activities in Chile have been supported through professional associations, institutional partnerships, and project-based resources, durable implementation requires stable funding and stewardship mechanisms. Training, coordination, data management, and community engagement cannot rely indefinitely on voluntary efforts or short-term enthusiasm. Sustainable pathways may include integration of SPEs competencies into formal primary care training curricula, alignment with national climate and health strategies, and linkage to public funding streams related to disaster preparedness, environmental surveillance, and health system resilience. However, these pathways remain developmental and require political commitment and institutional anchoring.

##### Ethical Risks, Stigma, and Vulnerable Populations

Finally, ethical considerations are central to the SPEs framework, particularly the risk that environmental health alerts may inadvertently stigmatise already vulnerable populations or neighbourhoods. This concern is especially relevant in contexts involving migrant communities, informal settlements, or areas characterised by poverty and environmental injustice. The SPEs model explicitly recognises this risk and emphasises safeguards such as equity-oriented framing of health messages, community co-design of communication strategies, and the avoidance of individual or group blame. By grounding environmental alerts in solidarity, prevention, and collective protection, SPEs can contribute to risk communication that empowers communities rather than reinforcing marginalisation.

Taken together, these challenges do not undermine the relevance of the SPEs approach, but rather delineate the conditions under which it can be credibly implemented and meaningfully evaluated. By explicitly acknowledging operational burdens, governance constraints, and ethical risks, the SPE framework is positioned as a realistic, reflexive, and context-sensitive model for strengthening environmental health action within primary health care.

### 8.5. Conceptual Monitoring and Evaluation Framework for Sentinel Physicians for the Environment

The development and implementation of Sentinel Physicians for the Environment (SPEs) require an explicit approach to monitoring and evaluation (M&E) in order to ensure transparency, learning, and accountability over time. Given the developmental and pilot-oriented nature of current SPE initiatives in Chile and Latin America, the framework proposed here is conceptual and prospective rather than evaluative. It is intended to guide future pilot studies and institutional learning, rather than to report measured performance outcomes.

A structured M&E approach for SPEs can be organised across three complementary levels: process, output, and outcome indicators. This distinction helps clarify what can be reasonably monitored in early implementation phases and what requires longer-term observation and system maturity.

**Process indicators** focus on the integration of SPE functions into routine primary care practice and governance mechanisms. Illustrative examples include the number of clinical consultations incorporating environmental anamnesis; the frequency of cluster reports generated by SPE-trained physicians; participation of SPEs in municipal or intersectoral coordination meetings; and the use of shared reporting tools linking primary care with epidemiological or environmental surveillance units.

**Output indicators** capture the immediate products of SPE activities and their translation into public-health action. Proposed indicators may include the number and scope of community risk advisories issued following SPE signal detection; the proportion of vulnerable patients (e.g., older adults, migrants, people with chronic conditions) with documented environmental exposure or vulnerability assessment; and the timeliness of information flow from clinical observation to municipal or regional notification during environmental or climate-related events.

**Outcome indicators**, by contrast, require longer-term observation and should be interpreted cautiously in early phases. Examples may include reductions in detection lag for climate- or environment-related health events compared with pre-SPE baselines; increased continuity of care during extreme events; and the proportion of municipal preparedness or response actions explicitly informed by SPE-generated clinical–environmental intelligence. These outcomes are contingent on system-level factors, governance arrangements, and data infrastructure, and therefore cannot be attributed to SPEs alone in the absence of rigorous evaluation designs.

All indicators presented here are explicitly proposed as illustrative and forward-looking metrics for future pilot evaluation. They are not intended to suggest that such indicators have already been measured or validated. Rather, this conceptual M&E framework demonstrates that the SPE model is evaluable in principle and provides a transparent foundation for future empirical assessment.

## 9. Conclusions

### 9.1. Key Messages from the Chilean Experience

The Chilean experience with Sentinel Physicians for the Environment shows that General Practitioners (GPs) can play a decisive role in strengthening community and health system preparedness and adaptive capacity. SPEs can enhance preparedness for climate-related disasters, reinforce early warning systems for vector-borne diseases, and reduce inequities by addressing the needs of vulnerable groups such as children and migrants. Rooted in local realities but aligned with One Health, Planetary Health, and the Sustainable Development Goals, the SPEs model highlights how primary care can evolve into a strategic driver of equity and sustainability.

### 9.2. Looking Ahead: From Training to Pilot Experiences

The next step will be to move from institutional proposals to concrete implementation. Pilot initiatives linked to the future National School of SPEs can provide testing grounds for new competencies in practice, combining clinical observation, environmental surveillance, and community engagement.

This forward-looking perspective shows how SPEs can evolve from individual training to systemic innovation, reinforcing adaptive capacity, reducing inequities, and embedding adaptive preparedness within health systems and communities.

These pilot initiatives will also generate empirical indicators for evaluation, addressing one of the main limitations identified in this paper and providing measurable outcomes for future research.

## Figures and Tables

**Table 1:** Pillar-by-pillar operationalisation of the Sentinel Physicians for the Environment (SPE) framework, distinguishing key operational constraints, reporting and information pathways, and proposed illustrative indicators for each core function.

SPE Pillar	Operational Constraints and Resource Requirements	Reporting and Information Flow	Proposed Illustrative Indicators (Future Pilot Evaluation)
**Surveillance**	Limited consultation time; lack of structured environmental fields in electronic health records; need for coordination between primary care and epidemiological services	Primary care consultation → documentation of environmental or exposure-related signals → municipal health offices → regional epidemiological surveillance units	Time elapsed between clinical cluster identification and notification; number of environment-related cluster reports generated by primary care
**Prevention**	Competing clinical priorities; need for patient engagement; limited time for preventive counselling; availability of local prevention services	Clinical encounter → preventive counselling and risk communication → referral to local health or social services	Proportion of high-risk patients receiving documented preventive counselling; documentation of environmental risk factors in primary care records
**Communication**	Risk of misinformation; need for coordinated institutional messaging; linguistic and cultural barriers within communities	SPE signal detection → municipal communication units → community risk advisories and public information channels	Number of community advisories issued following SPE signals; timeliness of risk communication during environmental or climate-related events
**Advocacy**	Political sensitivity of environmental health issues; institutional mandates; need to protect individual clinicians from personal exposure	Aggregated SPE observations → professional associations and public-health institutions → policy dialogue at municipal or regional level	Inclusion of SPE inputs in municipal or regional policy actions; frequency of institutional reports informed by SPE-generated evidence

## Data Availability

The original contributions presented in this study are included in the article. Further inquiries can be directed to the corresponding author(s).
